# Protocol for a Field Trial of a Hearing and Vision Support Intervention for People Living in Long‐Term Care in Australia

**DOI:** 10.1111/hex.70175

**Published:** 2025-02-10

**Authors:** Carly Meyer, Najwan El‐Saifi, Naomi Rose, Kasia Bail, Colette Browning, Dayna Cenin, Antonio Ahumada‐Canale, Megan Campbell, Tim England, Melanie Ferguson, Yuanyuan Gu, Reema Harrison, Chyrisse Heine, Lisa Keay, Sheela Kumaran, Iracema Leroi, Gerald Liew, Angelita Martini, Ralph Martins, John Newall, Smriti Raichand, Emma Scanlan, Hamid R. Sohrabi, Melinda Toomey, Johanna Westbrook, Piers Dawes

**Affiliations:** ^1^ School of Health and Rehabilitation Sciences The University of Queensland Brisbane Australia; ^2^ The University of Queensland Centre for Hearing Research (CHEAR) The University of Queensland Brisbane Australia; ^3^ Bolton Clarke Research Institute, Bolton Clarke Brisbane Australia; ^4^ Centre for Healthy Ageing, Health Futures Institute Murdoch University Perth Australia; ^5^ Centre of Ageing Research and Translation University of Canberra Canberra Australia; ^6^ Federation University Australia Mt Helen Australia; ^7^ Brightwater Research Centre Perth Australia; ^8^ Centre for the Health Economics, Research and Evaluation University of Technology Sydney Broadway Australia; ^9^ Freelance Consultant Sydney Australia; ^10^ Curtin University Perth Australia; ^11^ Macquarie University Centre for the Health Economy Sydney Australia; ^12^ Australian Institute of Health Innovation Macquarie University Sydney Australia; ^13^ Federation University Australia Ballarat and Gippsland Australia; ^14^ University of New South Wales Sydney Australia; ^15^ Trinity College Dublin Global Brain Health Institute Dublin Ireland; ^16^ University of Sydney Sydney Australia; ^17^ University of Western Australia Perth Australia; ^18^ Edith Cowan University Perth Australia; ^19^ Macquarie University Centre for Language Sciences Sydney Australia; ^20^ Hearing Australia Sydney Australia

## Abstract

**Introduction:**

Hearing and vision impairments are prevalent among residents in long‐term care settings. Hearing and vision impairments frequently go unsupported, affecting residents' quality of life and healthcare costs. This paper describes the protocol for a pre−post evaluation and process evaluation of a pragmatic sensory support intervention (SSI) that was developed with residents, informal caregivers and long‐term care workers.

**Methods and Analysis:**

A prospective pre−post‐intervention trial within long‐term care will be conducted, including three groups: residents (*n* = 87), informal caregivers (*n* = 87) and long‐term care workers (*n* = 40). Outcome measures include health‐related quality of life and well‐being measures relevant to each group measured at baseline, 3‐ and 6‐months post‐intervention. Health resource and sensory device utilisation will be captured from routine data and by direct observation. Qualitative interviews, including a representative sample of residents and informal caregivers, will be conducted as part of a simultaneous process evaluation. Generalised linear models and paired *t*‐tests will be used to evaluate the effects on residents' and caregivers' quality of life and sensory device use. The cost‐effectiveness of the intervention will be examined using within‐trial analysis, economic modelling and budget impact assessment. A process evaluation will use descriptive statistics and thematic analysis to assess the intervention's reach, adoption, implementation, acceptability, mechanisms of change and contextual influences.

**Conclusion:**

Hearing and vision impairments are common but under‐supported in older adults in long‐term care. This protocol involves a systematic evaluation of the effectiveness and implementation challenges of a pragmatic SSI to optimise hearing and vision function and improve the quality of life for long‐term care residents and their caregivers.

## 1 Introduction

Hearing impairment, vision impairment and dual sensory (i.e., both hearing and vision) impairment are highly prevalent among people resident within long‐term care settings [2, 3, 4, 5]. Among 4007 residents living in long‐term care across eight European countries, 32% had a dual sensory impairment, 12.3% had hearing impairment only (based on the InterRAI instrument for long‐term care facilities) [6] (REF), with hearing impairment defined as difficulty hearing (e.g., when a person speaks softly and is more than 6 feet away) to no hearing aid all, even with the use of hearing aids), and 19.5% had vision impairment (difficulty seeing [e.g., large print] to having no vision at all, even with the use of glasses or other visual aids) [2].

Approximately half of the residents wore glasses or used another visual appliance and 9% used hearing aids [2]. Sensory impairments exacerbate the impact of cognitive impairment or dementia, and for those living with dementia, management of hearing and vision impairment is more complex [7, 8, 9, 10]. Hearing and vision impairment both result in communication difficulties and difficulties reading text or understanding visual information. These functional difficulties can be masked by dementia or mistakenly attributed to cognitive impairment rather than recognised as a potentially treatable sensory impairment [9, 11].

It is important that sensory impairments are effectively managed in long‐term care settings as both result in activity limitations and participation restrictions, which may negatively impact overall well‐being. Both hearing and vision impairment separately are associated with loneliness [12, 13, 14, 15], anxiety [16, 17] and depression [17], with even worse outcomes for those with dual sensory impairment [17, 18].

Unfortunately, sensory impairments are underdiagnosed and poorly managed in long‐term care settings [19, 20, 21]. Reasons for poor management include a lack of knowledge among long‐term care staff regarding amplification devices [21], how to care and manage devices [22, 23], and an overall lack of training to use hearing and vision devices [22, 23, 24]; sensory needs not prioritised among staff [24, 25]; unclear referral pathways and difficulties accessing hearing and vision services [22, 26, 27, 28]; and a lack of suitable hearing and vision screening tools/protocols for people with dementia [29].

To address these challenges and support long‐term care staff to deliver high‐quality hearing and vision support to residents, we drew on behavioural science frameworks and principles [30, 31] to identify key needs and corresponding components of a sensory support intervention (SSI) and to understand barriers to implementation so that these could be addressed [32, 33]. The intervention aims to support long‐term care staff in (1) assisting residents with day‐to‐day device management and (2) communicating effectively with residents who have hearing and/or vision impairment [32, 33, 34]. Delivery of the intervention is supported by sensory champions identified from among long‐term care staff who will be responsible for identifying residents' hearing, vision and communication needs and preferences, increasing awareness of hearing and vision services available and carrying out an audit of residents' rooms and living areas to optimise them for sensory functioning (e.g., reducing background noise). Champions have proved to be effective in supporting long‐term care workers in relation to quality of care and improved resident health outcomes [35], including maintaining hearing aids and using communication strategies for residents with hearing impairment [36].

## 2 Research Questions

This study uses prospective pre−post‐intervention trial design. The primary research question (RQ) is:

What is the impact of the SSI on the quality of life of residents and their informal caregivers (i.e., unpaid care provided by family, close relatives and friends) [37]? This RQ will be explored through standardised questionnaires (e.g., the Health Utilities Index 3 [HUI‐3] [38] and the Quality of Life‐Aged Care Consumers questionnaire [QOL‐ACC] [39], described below).

Secondary research questions include:

What is the impact of the SSI on care workers' (formal caregivers of people living in long‐term care) use of sensory support behaviours? This RQ will be explored through direct observation and questionnaires.

What is the impact of the SSI on residents' use of hearing and vision devices, quality of resident‐staff communication, well‐being, sensory function and behaviour? This RQ will be explored through direct observation, self (or proxy) report questionnaires and professional caregiver report.

What is the impact of the SSI on informal caregivers' well‐being and relationship with residents? This RQ will be explored through questionnaires.

What is the cost‐effectiveness of the SSI compared to standard care? This RQ will be explored through direct observation and questionnaires.

A simultaneous process evaluation is also planned to address the following research questions:

To what extent was the SSI considered acceptable to care workers, residents and informal caregivers and implemented as intended? Acceptability will be explored through a post‐intervention survey and individual structured qualitative interviews, and implementation will be evaluated based on inspection of daily task sheets that record the daily duties of care workers.

What were the mechanisms through which the SSI achieved/did not achieve impact? This RQ will be explored using individual, semi‐structured, in‐depth interviews.

How did contextual factors impact the implementation of the SSI? This RQ will be explored by analysing the written log completed by the sensory champion, supplemented by a structured qualitative interview.

## 3 Methods and Analysis

### 3.1 Sample

Participants will include three groups: (1) people living in long‐term care, (2) informal caregivers of people living in long‐term care and (3) long‐term care workers. All participants will be recruited from a single long‐term care setting in Perth, Western Australia, who we partnered with to develop the intervention. All people living at the participating long‐term care setting over the age of 65 will be eligible to take part, irrespective of their sensory or cognitive functioning. Informal caregivers will be eligible to participate if they are aged 18 years or older and are a family member or friend of a resident participating in this study. Informal caregivers will be excluded if they are not in regular contact with the resident. Long‐term care workers will be eligible to participate if they currently work in the participating long‐term care setting and are involved in the provision of daily personal care to residents participating in this study.

### 3.2 SSI

The SSI is twofold. First, we will be intervening directly with long‐term care workers to support them to carry out three target behaviours: (1) assist residents with day‐to‐day hearing and vision device use, (2) use appropriate communication and (3) mute TV/radio at the bedside and during mealtimes when communicating. These behaviours are fully specified in Table 1. We will be carrying out a multifaceted intervention that addresses the key barriers and enablers identified in our exploratory Phase 1 research [32, 33, 34], as described in Table 2. Second, one or two staff at each participating site will be assigned a ‘sensory champion role’. Previous studies have shown that designating one or more staff members as ‘champions’ in long‐term care facilities and providing them with additional training and responsibilities can enhance their competence in managing related issues, improve the quality of care and positively impact the health outcomes of residents [35]. A subsequent study reported that designated ‘hearing champions’ were effective in supporting staff in relation to the maintenance of hearing aids and the use of communication strategies for residents with hearing impairment [36]. The sensory champion may be a registered nurse, care worker or therapy assistant, and will be employed in this capacity for 2−3 days per week for 3 months at each site. A description of the tasks completed by the sensory champion is outlined in Table 3.

**Table 1:** Full specification of staff behaviours targeted in the sensory support intervention.

Behaviour	Full specification
Assist residents with day‐to‐day hearing/vision device use	Each morning, check glasses are on and clean (glasses wearers) Each morning, check that hearing aids are on, inserted correctly and on correct ears and working (HA users) Each evening, check hearing aids and personal listening devices are removed, wiped clean and stored safely and batteries are conserved (on charge/battery door open) (HA/ALD users) Each week, check frames are well aligned and in good order (e.g., screws tight, nose pads intact, no corrosion of metal frames or fragmentation of plastic) (glasses wearers)
Use appropriate communication	Gain attention Speak face‐to‐face Speak clearer, nearer, slower Make text brighter, bigger, bolder
Make small changes to residents' environment	Request permission to mute TV or radio before talking to residents in their room Turn off music/TV in communal areas during mealtimes

Abbreviations: ALD = assistive listening device, HA = hearing aid.

**Table 2:** Summary of sensory support intervention for long‐term care workers.

Intervention component	Barrier that is being addressed	Description
Mastering sensory support Education and training, delivered by lead sensory therapist Training materials were developed by experts in audiology and optometry, supplemented by resources from our international collaborators and leading organisations such as Hearing Australia.	Knowledge and skills related to: Device management: This includes understanding different types of sensory devices, caring for these devices, inserting and removing hearing aids and ensuring they are working properly.	Tailored to each centre, a 60 min education and training session will be held on multiple days, either before or shifts. Training will be delivered by a member of staff at each centre. These sessions will cover the following topics: Device management. Effective communication with residents who have sensory impairment and/or dementia. Support good sensory cognitive health: support device use, reduce background noise and support the behaviour of residents with dementia while fostering an environment that respects their choices and preferences.
	Interpersonal skills: The ability to respond effectively to residents' negative reactions and behaviours, particularly those exhibited by residents with dementia. Background noise impact: Knowledge of how background noise affects residents with sensory impairment.	
Sensory support goal setting	Long‐term care staff are task‐orientated.	Task list: Integrating sensory support items (support device use, effective communication and reduction of background noise) into the staff's daily task list. Staff recognition: ‘Sensory support star’.
Sensory support station	Devices are not always accessible as they are easily lost. Devices are not labelled.	Designate specific areas within residents' rooms and communal areas as dedicated sensory stations equipped with sensory devices (e.g., hearing aids, glasses), cleaning and maintenance kit. Labelling options will be discussed with residents.
Sensory support prompt Use of visual cues and reminders in strategic locations	Forgetfulness to enact sensory support behaviours.	Tailored to each centre, use a physical prompt (e.g., a poster placed on the back of the main door of the resident's room).

**Table 3:** Summary of sensory champion role.

Intervention component	Description	Duration
Understand hearing and vision function	Baseline data pertaining to hearing and vision function collected by the research team will be shared with the sensory champion. NB: if the intervention is shown to be effective, we will advocate for screening measures to be incorporated into routine care.	NA (incorporated into data collection)
Sensory needs analysis	The sensory champion will extract hearing and vision records from the resident's chart (if available), confirm they are up‐to‐date, and discuss their preferences regarding hearing and vision device use and communication strategies.	Approximately 60 min
Provide information about intervention pathways	Based on the sensory needs analysis, residents will be provided with information about potential intervention pathways (if required).	Approximately 30 min
Sensory support station and sensory support prompt discussion	Discussions with residents about their preference for a designated area to store and maintain all their sensory support devices and related items. Additionally, discuss whether they are agreeable to placing visual prompts to remind staff of sensory support‐related tasks.	Approximately 30 min
Environmental audit	Residents' rooms will be audited to ensure they are optimally set up for hearing and vision impairment.	Approximately 30 min
Device troubleshooting and repair	Aged care staff will be informed that they can contact the Sensory Champion should a resident's hearing/vision aids stop working. The Sensory Champion will try to troubleshoot the problem (based on their training) and arrange for repair if required.	As needed
Referral to hearing and vision specialists and GPs	If requested, the Sensory Champion will facilitate referral to appropriate hearing, vision or medical services (e.g., for ear wax management and supported administration of eye drops).	As needed

All residents with dementia will be encouraged to have a family member accompany them to assessment appointments and appointments with a sensory champion. For residents with dementia who may revert to their first language, we will seek input from family members, who may be able to translate, use gestures to communicate or utilise translation apps. We will also consult with long‐term staff who care for the resident to identify communication preferences.

### 3.3 Sample Size Calculation

Approximately 87 residents will be recruited at baseline. The evaluation is powered to detect a standardised effect size of *d* = 0.3 (because hearing/vision interventions are associated with small‐to‐medium effects on health‐related quality of life outcomes in the primary treatment group (i.e., residents) [40, 41, 42]. Assuming a correlation of 0.6 between baseline and 12‐week follow‐up Health Utility Index‐3 (HUI‐3) scores and an attrition rate of 20% at follow‐up (a conservative estimate based on the 12%–15% rates observed in previous similar studies) we will need to recruit 87 residents at baseline to achieve 80% power to detect the effect size at the two‐sided 5% level of significance, according to estimates generated by G*Power software [43].

### 3.4 Baseline Measurement

#### 3.4.1 Demographics and Health Status

Information pertaining to residents' age, sex, ethnicity, time in long‐term care and level of care need will be extracted from their long‐term care records. Formal and informal caregivers will be asked to complete a purposefully developed questionnaire to document their age, sex, ethnicity and educational level.

#### 3.4.2 Sensory and Cognitive Functioning

Residents will complete a purposefully developed questionnaire that will ask about their history of hearing, vision and cognitive management before completing objective screening assessments. Both ears will be examined using a Welch Allyn pocket LED otoscope to detect impacted ear wax; where this is present, the client will be referred to a health professional for ear wax removal. Hearing ability will subsequently be screened using HearX HearCheck pure‐tone air‐conduction audiometric testing using a Samsung Galaxy A04e phone with Sennheiser HD280 Pro headphones. Each ear will be tested at 0.5, 1, 2 and 4 kHz, with hearing loss identified if screening thresholds are greater than 35 dB HL in the better ear. Vision screening will include visual acuity screening using PEEK Acuity [44], Spaeth/Richman contrast sensitivity test (SPARCS) [45], central vision testing using an Amsler grid and visual field‐testing using confrontation testing. PEEK acuity will be conducted using a Samsung phone with a screen measuring 13.5 cm by 6.5 cm. PEEK acuity scores are provided in standard units of Snellen—including metric (6/6) and imperial (20/20). SPARCS contrast sensitivity will be conducted using a portable external monitor measuring 17.3 inches. SPARCS evaluates contrast sensitivity across five areas of the visual field: the left upper quadrant, left lower quadrant, right upper quadrant, right lower quadrant and central area. Log‐based scores for each of the five testing regions are scaled from 0 to 20, with the highest possible SPARCS score being 100.

Global cognitive functioning will be evaluated using the hearing or vision impairment version of the Montreal Cognitive Assessment (MoCA) [46, 47]. In the hearing impairment version, three auditory items were substituted with written items [47]; and in the vision impairment version, four visual items were substituted with spoken items. A cut‐off of 24/30 is indicative of cognitive impairment for both versions [46, 47]. Participants with dual sensory impairment will complete the version developed for people with vision impairment with the aid of a personal amplifier, if not wearing hearing aids.

Sensory and cognitive functioning assessment will be conducted by three research assistants with a background in health research and having been trained in hearing and vision assessments by qualified audiologists and optometrists. Additionally, the research assistants will be certified to administer and score the MoCA.

### 3.5 Outcome Measures

To evaluate intervention effectiveness, participants will undergo assessment at baseline, throughout the intervention, and at 3 and 6 months post‐intervention. Data will be derived from a variety of methods, including self‐ or proxy‐report questionnaires; long‐term care records; participant observation and qualitative interviews.

#### 3.5.1 Primary Outcome

Residents' quality of life will be measured using the interviewer‐administered versions of the HUI‐3 40Q [38] questionnaire with 1‐week recall, and the Quality of Life‐Aged Care Consumers (QOL‐ACC) [39] instrument. The HUI‐3 captures the sensory quality of life attributes and therefore will be more sensitive to the effect of the intervention. The interviewer‐administered version of the HUI‐3 was chosen as some participants will likely have mild cognitive impairment or dementia and therefore many may be unable to complete a self‐report measure. The QOL‐ACC will also be used to capture the quality of life as the HUI‐3 has shown inconsistencies in people with dementia. The QOL‐ACC has been validated both for people with dementia and people living in long‐term care [48] and is routinely collected by the Australian Government for long‐term care residents. We will measure QoL using both self‐assessed and proxy‐assessed versions of both HUI‐3 and QOL‐ACC to ensure completeness, where proxy‐assessment will be completed by the informal caregivers or long‐term care workers where an informal caregiver is not available. QOL‐ACC and HUI‐3 responses will be used to estimate utilities with an Australian algorithm [49, 50]. Utilities will be used to estimate quality‐adjusted life years (QALYs), which combine the utility of a health state with the time spent on that health state. QALYs will be used as the benefit measure in the cost‐effectiveness analyses. To capture the impact of the SSI on the duration of daily care delivery, a research assistant will use field notes to record the duration of daily care delivery based on direct observation of care workers entering and leaving the resident's room during baseline and post‐intervention periods.

Informal caregivers' quality of life will be evaluated using the 5‐item EQ‐5D‐5L [51], which evaluates five dimensions, including mobility, self‐care, usual activities, pain/discomfort and anxiety/depression. Higher scores reflect poorer quality of life. Utilities will be estimated using an Australian algorithm [51, 52].

#### 3.5.2 Secondary Outcomes

To evaluate the impact of the SSI on long‐term care workers' use of sensory support behaviours (RQ2), care workers' hearing aid and glasses management knowledge and skills will be assessed, sound and light measurements will be taken and task lists that record the daily duties of care staff will be reviewed. Hearing aid and glasses management knowledge and skills will be assessed at three time points (baseline, after the training intervention and after 3 months follow‐up) using modified versions of the Hearing Aid Skills Knowledge assessment and the Glasses Skills Knowledge assessment [53, 54]. Background noise and lighting levels will be recorded using observational measures including a modified version of the Mealtime Scan [55] as well as sound and light measurements in residents' rooms, corridors and communal dining areas. The Mealtime Scan is a tool to quantify the overall dining environment by assessing the physical and social environments as well as person‐centred care practices [55]. For the current study, the Mealtime Scan was adapted to focus on social interactions and sound measurement during the meal. Four observations will be made at baseline, 3 months and 6 months. Sound and light levels will be measured using a Protech Pro Sound Level Metre and a Protech Professional 400 K Lux Metre. In common areas, the average of three measurements will be recorded at each of the four central points of a square grid covering the extent of the room: the top‐left, top‐right, bottom‐left and bottom‐right. In residents' rooms, two light measurements will be taken: one measurement of ambient light levels and another from a location where the resident typically performs activities (e.g., reading in bed, armchair or table). Long‐term care workers' task lists will be reviewed to verify completion of sensory support tasks, including device management, use of communication strategies and background noise reduction over a period of 3 days at baseline and post‐intervention.

To evaluate the impact of the intervention on residents' use of hearing and vision devices, quality of resident‐provider communication, well‐being, sensory function and behaviour (RQ3), we will employ direct observation, self (or proxy) report questionnaires and professional caregiver report. The use of hearing and vision devices will be measured using direct observation and resident self‐report. Residents will complete the International Outcome Inventory‐Hearing Aids (IOI‐HA) if they own hearing aids [56]. Items on the IOI‐HA are scored on a 5‐point Likert scale from left (worst) to right (best), with higher scores representing better hearing aid outcomes. Resident‐provider communication will be measured using two subscales of the Patient Evaluation of Emotional Care During Hospitalisation (PEECH) questionnaire [57, 58] capturing ‘level of personal value’ (10 items) and ‘level of connection’ (3 items). Items are measured on a 4‐point scale (3 = all, 2 = most, 1 = some, 0 = none) with higher scores reflecting better patient‐provider communication. Overall well‐being will be measured using the 20‐item Geriatric Anxiety Inventory [59] and 8‐item Geriatric Depression Scale (nursing home version) [60]. Participants are asked to respond with yes/no to a series of statements that ask about symptoms of anxiety and depression. Functional and social impacts of hearing loss will be evaluated using the 5‐item Social Isolation Measure using an 11‐point Likert scale [61] and the 10‐item Revised Hearing Handicap Inventory—Screening (or proxy version) where each item is assigned a score of 4, 2 or 0 [62]. Both measures ask about the social and emotional impacts of hearing loss, with higher scores being indicative of more perceived hearing‐related activity limitations and participation restrictions. Vision function will be measured using the 20‐item Veterans Affairs Low Vision Visual Functioning Questionnaire–short form (LV‐VFQ‐20) (or proxy version) [63], which asks about the impact of corrected vision in four domains: reading, visual motor, visual information processing and mobility. Each item is measured on a 5‐item scale: ‘not difficult’, ‘slightly/moderately difficult’, ‘extremely difficult’, ‘impossible’ and ‘do not do it for nonvisual reasons’. The presence of responsive behaviours will be evaluated by professional care staff using the Neuropsychiatric Inventory–care home version (NPI‐NH) [64]. Lastly, routinely collected data pertaining to the National Aged Care Quality indicators (e.g., medication management, falls and major injury) will be extracted from participants' long‐term care records.

To evaluate the impact of the SSI on informal caregivers' well‐being and relationships with the residents (RQ4), overall well‐being will be measured using the 12‐item General Health Questionnaire (GHQ‐12) [65], and relationship satisfaction will be measured using the 7‐item Burns Relationship Satisfaction Scale [66]. Higher scores reflect higher levels of mental distress and greater relationship satisfaction, respectively.

#### 3.5.3 Process Evaluation

We will carry out a process evaluation in the month following the intervention at each participating site as outlined below.

To address RQ6, implementation will be measured using the following dimensions of RE‐AIM (https://re-aim.org/) (alongside effectiveness measures described as part of the primary evaluation) [67]:

- Reach: Percentage of the target population who participate, and differences in demographics between people who do participate and those of the overall target population (e.g., age, sex, cognitive functioning).
- Adoption: Percentage of staff invited who participate, and characteristics of long‐term care workers participants versus nonparticipating staff (e.g., role, years of experience).
- Implementation: Record of adaptations made to the SSI during the study, and long‐term care workers' adherence to intervention components (e.g., completion of sensory champion tasks, attendance at training sessions).

Perceived acceptability of the intervention will be evaluated using a combination of a post‐intervention survey and individual, structured interviews with a subsample of approximately 20 participating long‐term care workers. The survey and interview topic guide will be informed by the Theoretical Framework of Acceptability [68, 69].

Mechanisms of impact (RQ7) will be explored using individual, semi‐structured, in‐depth interviews conducted by the sensory Lead Therapist, a speech pathologist with experience in conducting qualitative interviews. These interviews will involve a subsample of approximately 20 participating long‐term care workers to understand the extent to which the SSI achieved behaviour change via the mechanisms proposed in our earlier behavioural analysis (e.g., our intervention resulted in improved knowledge of (1) how to insert hearing aids, (2) who uses hearing/vision devices, which subsequently results in increased use of day‐to‐day device management).

Contextual influences (RQ8) will be explored using a written log maintained by the Sensory Champion(s) and individual, semi‐structured interviews at the conclusion of the study. The contextual domains, as defined by the Context and Implementation of Complex Interventions (CICI) framework [70] include: geographical (e.g., infrastructure, availability of resources); epidemiological (e.g., level of care needs within the long‐term care home, lockdowns due to disease outbreaks); socio‐cultural, ethical and legal (e.g., organisational culture, governance); socioeconomic (e.g., residents' access to hearing and vision services/devices); and political (e.g., policy changes, workforce issues, long‐term care home closures) domains.

### 3.6 Data Analysis Plan

We will apply methods (e.g., Bonferroni correction) to control for family‐wise error rates for each research question. Data analysis will be conducted with SPSS [71].

Two generalised linear models will be applied to evaluate the impact of the SSI on the quality of life of residents and their informal caregivers. For both, the dependent variables will be the individual domains of quality of life (resident or informal caregiver) and the independent variable will be time. Potential covariates will include demographic variables, (resident) health status, (resident) cognitive and sensory functioning, (resident) use of hearing and vision devices and use of long‐term care workers' sensory support behaviours.

Paired *t*‐tests (or other repeated measures test) will be used to examine pre‐/post differences in long‐term care workers' use of sensory support behaviours.

A series of generalised linear models will be used to evaluate the impact of the SSI on residents' use of hearing/vision devices, quality of patient‐provider communication, well‐being, sensory function and behaviour. For each model, the independent variable will be time and potential covariates will include demographic variables, health status, cognitive and sensory functioning and use of long‐term care workers' sensory support behaviours.

Two generalised linear models will be applied to evaluate the impact of the SSI on informal caregivers' well‐being and relationship with the resident. For each model, the independent variable will be time. Potential covariates will include demographic variables, resident health status, resident cognitive and sensory functioning and use of long‐term care workers' sensory support behaviours. The nested structure of the data will be taken into consideration in the modelling.

To evaluate the cost‐effectiveness of the SS intervention compared to standard care (RQ5), we will report a within‐trial analysis, economic modelling and a budget impact analysis. The within‐trial analysis will include two components: (1) the health benefits associated with QoL outcomes for participants, as well as informal caregivers (RQ1), and (2) costs associated with intervention and healthcare resources using a micro‐costing study. We will estimate the intervention cost by recording the time spent by long‐term care workers on supporting residents' hearing and vision needs over 5 days, and extrapolate it over the relevant time horizon. Healthcare resource use will be measured at baseline and at a 3‐ and a 6‐month follow‐up using the Resource Utilisation in Dementia (RUD) Lite [72, 73], a standardised tool for collecting resource use data in dementia care. Since the tool was originally developed for community settings, only the section related to patient healthcare resource utilisation will be used to fit long‐term care settings. For informal caregivers, we will use the three sections of the RUD Lite concerning caregivers detailing primary caregiver description, time and work status.

Unit prices will be sourced from the corresponding Medicare Benefits Scheme items, Pharmaceutical Benefits Scheme items, the National Hospital Cost Data Collection [74] and other relevant sources.

We will estimate the lifetime cost‐effectiveness of the intervention using economic modelling (e.g., Markov modelling). We will conduct a literature review to inform the model structure and parameters not sourced from the intervention study. We will also report a budget impact analysis to determine the financial implications of implementing the SSI for the federal government. We will report the results of the cost‐effectiveness analyses according to the Consolidated Health Economic Evaluation Reporting Standards (CHEERS) checklist [75].

The economic evaluation outcome will be the incremental cost‐effectiveness ratio (ICER), which will determine whether the intervention is cost‐effective using Australia's implicit cost‐effectiveness threshold [76]. The ICER represents the incremental cost associated with a unit gain of health outcome of interest (e.g., a QALY) between two interventions.

The equation for the ICER is represented as:

$$\mathrm{ICER}=\frac{C_{1}-C_{0}}{E_{1}-E_{0}}$$

where *C*_1_ and *C*_0_ are the costs associated with the long‐term care model and the usual care, respectively, while *E*_1_ and *E*_0_ are the effectiveness estimates (QALYs) of the of the long‐term care model and the usual care, respectively. The numerator and denominator for the ICER calculation will be estimated using generalised linear models accounting for data distribution and covariates. Sampling uncertainty will be handled by nonparametric bootstrap sampling. Bootstrap iterations will be plotted in the cost‐effectiveness plane and cost‐effectiveness acceptability curves.

For the economic model, uncertainty around the lifetime cost‐effectiveness results will be explored using deterministic and probabilistic sensitivity analysis.

For the budget impact analysis, we will report the cost to the federal government in Australian dollars. We will estimate this cost to the Australian healthcare system using projected utilisation estimates of SSI in Australia in the long‐term care setting over a relevant time horizon.

RQ6‐8: Descriptive statistics will be used to examine reach, adoption, implementation and acceptability. The qualitative interviews will be transcribed verbatim and analysed using mixed deductive‐inductive thematic analysis [77] to explore (1) the acceptability of the intervention to residents, long‐term care workers and informal caregivers, (2) the mechanisms through which staff behaviour change occurred and (3) the impact of contextual factors on implementation. Coding frameworks will be developed a priori based on the Theoretical Framework of Acceptability [69], the Capability, Opportunity, and Motivation Model of Behaviour (COM‐B) [30, 31] and the CICI framework [70]. After thoroughly reading interview transcripts, meaning units will be assigned to the most appropriate theoretical construct after which subthemes will be identified inductively using Braun and Clarke's methodology [78]. Rigour will be enhanced through regular peer consensus meetings between two coders performing independent double coding and other members of the research team. Qualitative results will be reported in line with the consolidated criteria for reporting qualitative research (COREQ).

### 3.7 Patient and Public Involvement

Four informal caregivers or people with lived experience of hearing or vision impairment provided feedback, which was incorporated into the study information sheets and consent forms. Their suggestions included language simplification, correcting typographical errors and making minor wording changes to enhance clarity. Informal caregivers will also be consulted to provide iterative feedback on qualitative analysis to aid in contextualising the findings in the ‘real world’, as well as advise on public dissemination of the study findings. Consultations will be conducted via email to gather feedback on written materials, while in‐person meetings or video conferences will be used for more detailed, in‐depth discussions. The study output will include structured reporting of patient and public involvement as per Guidance for Reporting Involvement of Patients and the Public‐2 (GRIPP2) recommendations [1].

## 4 Ethics and Dissemination

This study has been approved by the University of Queensland Human Research Ethics Committee B (2023/HE001515) and ratified by collaborating institutions. The results from this study will be presented at both national and international conferences, disseminated to lay and scientific audiences using social media outlets and the project website (https://sense-cog.eu/), and submitted to peer‐reviewed journals for publication. Deidentified data will be stored in an online data repository (i.e., University of Queensland Espace) so they are accessible to other researchers.

## 5 Discussion

This paper has described a protocol for the evaluation of an SSI that aims to improve hearing and vision outcomes for older people living in long‐term care by supporting the practice of long‐term care workers and improving long‐term care environments. This protocol entails a prospective pre−post‐intervention trial conducted within a long‐term care setting incorporating pre‐intervention assessments (baseline) and at 3 and 6 months post‐intervention. Concurrently, a process evaluation will be conducted during the trial to assess implementation, mechanisms of impact and contextual factors influencing the intervention's effectiveness.

The protocol was developed by an interdisciplinary team with expertise in co‐designing complex intervention, implementation science, long‐term care, behaviour change, health economics, health professionals, hearing and vision care and dementia. This diversity of expertise ensures that multiple perspectives are considered so that the evaluation will provide both information about the benefits of the SSI and the challenges and opportunities for implementation. The evaluation of the multidisciplinary SSI is aligned with the Medical Research Council guidance for process evaluation of complex interventions [79]. The use of both qualitative and qualitative methodology facilitates insights into the benefits of the intervention and contextual factors that may not be captured by quantitative measures alone. Evidence about the cost‐effectiveness and acceptability of the intervention will inform large‐scale implementation to improve the quality of care provided to older Australians and optimise health outcomes.

Limitations of this study include involving a single provider long‐term care setting in one country (Australia), which may limit the generalisability of the results. However, outcomes from this study would be relevant for any countries with similar models of aged care. The sample size is relatively small; however, the estimated number of participants should be parsimonious but at the same time sufficient to detect change in effect with precision.

## 6 Conclusion

Hearing and vision impairments are common but under‐supported in older adults living in long‐term care. This protocol involves a systematic evaluation of the effectiveness and implementation challenges of a pragmatic SSI to optimise hearing and vision function and improve the quality of life for long‐term care residents and their caregivers. Our goal is that relatively simple changes in awareness and practice for aged care staff are achievable and will make a meaningful difference in the lives of older Australians.

## Author Contributions

**Carly Meyer:** conceptualisation, writing–original draft, project administration. **Najwan El‐Saifi:** conceptualisation, writing–review and editing, project administration. **Naomi Rose:** conceptualisation, writing–review and editing. **Kasia Bail:** conceptualisation, writing–review and editing. **Colette Browning:** conceptualisation, writing–review and editing, funding acquisition. **Dayna Cenin:** conceptualisation, writing–review and editing. **Antonio Ahumada‐Canale:** conceptualisation, writing–review and editing. **Megan Campbell:** conceptualisation, writing–review and editing. **Tim England:** conceptualisation, writing–review and editing, funding acquisition. **Melanie Ferguson:** conceptualisation, writing–review and editing. **Yuanyuan Gu:** conceptualisation, funding acquisition, writing–review and editing. **Reema Harrison:** conceptualisation, funding acquisition, writing–review and editing. **Chyrisse Heine:** conceptualisation, writing–review and editing, funding acquisition. **Lisa Keay:** conceptualisation, writing–review and editing, funding acquisition. **Sheela Kumaran:** conceptualisation, writing–review and editing. **Iracema Leroi:** conceptualisation, writing–review and editing, funding acquisition. **Gerald Liew:** conceptualisation, writing–review and editing, funding acquisition. **Angelita Martini:** conceptualisation, writing–review and editing, funding acquisition. **Ralph Martins:** conceptualisation, writing–review and editing, funding acquisition. **John Newall:** conceptualisation, writing–review and editing. **Smriti Raichand:** conceptualisation, writing–review and editing. **Emma Scanlan:** conceptualisation, writing–review and editing. **Hamid R. Sohrabi:** conceptualisation, funding acquisition, writing–review and editing. **Melinda Toomey:** conceptualisation, writing–review and editing. **Johanna Westbrook:** conceptualisation, writing–review and editing, funding acquisition. **Piers Dawes:** conceptualisation, funding acquisition, writing–review and editing.

## Conflicts of Interest

The authors declare no conflicts of interest.

## Data Availability

The data that support the findings of this study are available on request from the corresponding author. The data are not publicly available due to privacy or ethical restrictions.
